# miRNA-like duplexes as RNAi triggers with improved specificity

**DOI:** 10.3389/fgene.2012.00127

**Published:** 2012-07-12

**Authors:** Juan G. Betancur, Mayuko Yoda, Yukihide Tomari

**Affiliations:** ^1^Institute of Molecular and Cellular Biosciences, The University of Tokyo,Tokyo, Japan; ^2^Department of Medical Genome Sciences, The University of Tokyo, Tokyo, Japan

**Keywords:** Argonaute, RNA-induced silencing complex, RISC, asymmetry

## Abstract

siRNA duplexes, the most common triggers of RNA interference, are first loaded into an Argonaute (Ago) protein and then undergo unwinding via passenger strand cleavage, which requires the slicer activity of the Ago protein. In mammals, only Ago2 out of the four Ago proteins possesses such slicer activity. In contrast, miRNA/miRNA* duplexes often contain central mismatches that prevent slicer-dependent unwinding. Instead, mismatches in specific regions (seed and 3′-mid regions) promote efficient slicer-independent unwinding by any of the four mammalian Ago proteins. Both slicer-dependent and slicer-independent unwinding mechanisms produce guide-containing RNA-induced silencing complex (RISC), which silences target mRNAs by cleavage, translational repression, and/or deadenylation that leads to mRNA decay. In this review, we summarize our current knowledge of the RISC assembly pathways, and describe a simple method to rationally design artificial miRNA/miRNA*-like duplexes and highlight its benefits to reduce the unwanted “off-target” effects without compromising the specific target silencing activity.

## SMALL RNAs AND RISC

MicroRNAs (miRNAs) and small interfering RNAs (siRNAs) are the best-characterized small RNAs, which play crucial roles in post-transcriptional gene silencing. miRNAs are encoded in the genome and regulate various biological processes. Nowadays, the number of registered human miRNA genes has reached more than 1,000 and it is suggested that miRNAs are involved in various human diseases. On the other hand, siRNAs are derived from exogenous or endogenous double-stranded RNAs (dsRNAs) and act as a defense against viruses and other invasive nucleic acids (Carthew and Sontheimer, 2009; Ghildiyal and Zamore, 2009; Kim et al., 2009). In mammals, however, endogenous siRNAs have been reported only in mouse oocytes and ES cells (Babiarz et al., 2008; Tam et al., 2008; Watanabe et al., 2008; Song et al., 2011). Methods of target gene knockdown using siRNAs have been established and are used extensively in research as a convenient tool. There are also ongoing clinical trials using small RNAs as therapeutic agents (Burnett et al., 2011).

To exert their functions, siRNAs and miRNAs need to form an effector ribonucleoprotein complex, termed RNA-induced silencing complex (RISC). Because small RNAs only act as guide molecules to direct RISC to target mRNAs in a sequence-dependent manner, they can regulate the expression of their targets only after they form RISC. Thus, RISC assembly is a key process in small RNA-mediated gene silencing, and understanding of the assembly mechanisms is important for the development and improvement of therapeutic and research tools. The core component of RISC is a member of the Argonaute (Ago) subfamily of proteins, of which there are four in mammals (Ago1–4) and two in flies (Ago1 and 2). Each of the four mammalian Ago proteins can induce translational repression and/or deadenylation that leads to mRNA decay, but only Ago2 can endonucleolytically cleave target mRNAs when the sequence of the small RNA is extensively complementary to the target mRNA (Hutvagner and Simard, 2008; Carthew and Sontheimer, 2009; Ghildiyal and Zamore, 2009; Siomi and Siomi, 2009).

Although the biogenesis of miRNAs and siRNAs is different, both pathways result in the production of ~21–22 nt small RNA duplexes from their precursors (Carthew and Sontheimer, 2009; Ghildiyal and Zamore, 2009; Kim et al., 2009; Siomi and Siomi, 2009), known as miRNA/miRNA* duplexes and siRNA duplexes, respectively. These small RNA duplexes are assembled into Ago proteins and form RISC (Tabara et al., 1999; Hammond et al., 2001) in a process that consists of at least two steps: RISC loading and unwinding. In the RISC loading step, small RNA duplexes are first incorporated into Ago proteins as dsRNAs. The two strands are then separated into single-strand RNAs (ssRNAs) within the Ago proteins (unwinding). During unwinding, one strand (the passenger strand) is discarded, and only one strand (the guide strand) is retained in mature, functional RISC (Matranga et al., 2005; Miyoshi et al., 2005; Rand et al., 2005; Leuschner et al., 2006; Kawamata et al., 2009; Yoda et al., 2010). The strand of a miRNA duplex that is more frequently selected as guide strand is known as the miRNA strand whereas the opposite – and more likely to be discarded and degraded – strand is known as the miRNA* strand.

## RISC LOADING

**FIGURE 1:**
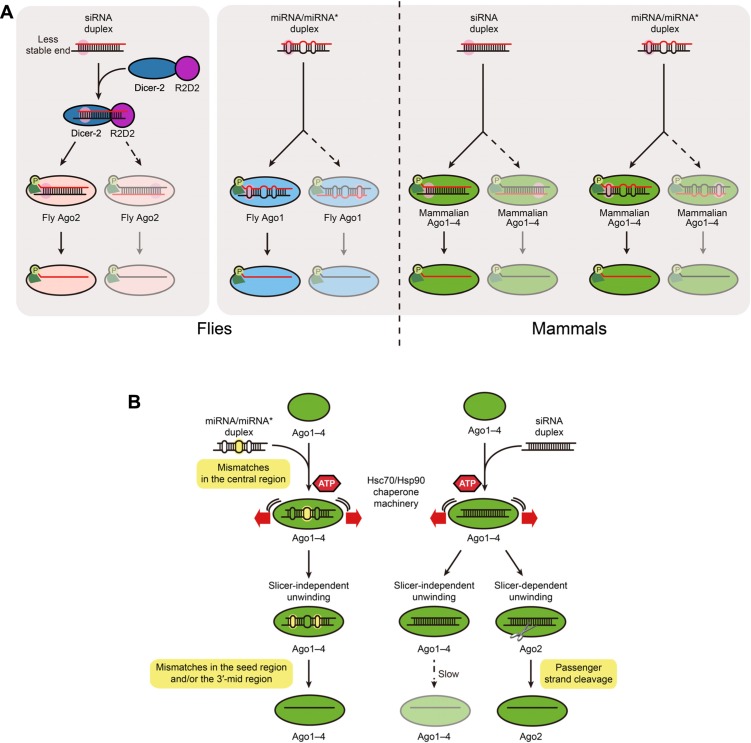
(A) miRNA/miRNA* and siRNA duplexes are functionally asymmetric. In both flies and mammals small RNA duplexes are preferentially loaded into Ago proteins with their less stable end (pink) toward the phosphate-binding pocket of the protein (light green), which results in the selection of the red strand as the guide strand. If the duplex is occasionally loaded in the opposite orientation, the black strand functions as the guide. The more asymmetric the duplex is, the more likely the red strand is exclusively selected as the guide. Besides Ago proteins themselves and the Hsc70/Hsp90 chaperone machinery **(B)**, no other factors are known to be required for asymmetric assembly of mammalian Ago1–4-RISC or fly Ago1 RISC. In contrast, in flies the Dicer-2/R2D2 heterodimer senses the asymmetry of siRNA duplexes and such binding is a prerequisite for Ago2-RISC assembly. **(B)** In mammals, central mismatches are preferred for RISC loading, whereas mismatches in the seed and 3′-mid regions promote slicer-independent unwinding. Such mismatches are highlighted in yellow. Duplex loading requires ATP and the Hsc70/Hsp90 chaperone machinery, while both slicer-dependent unwinding and slicer-independent unwinding do not.

In mammals, all four Ago proteins are known to incorporate both miRNA and siRNA duplexes (Liu et al., 2004; Meister et al., 2004; Azuma-Mukai et al., 2008; Su et al., 2009; Yoda et al., 2010). Importantly, the orientation in which small RNA duplexes are loaded into Ago determines which of the two strands of the duplex will remain incorporated in RISC (Tomari et al., 2004b; Matranga et al., 2005; Miyoshi et al., 2005; Rand et al., 2005; Leuschner et al., 2006). Such selection of the guide strand is not random, but rather asymmetric, and depends mainly on the thermodynamic stability of the ends of the duplex. The strand with its 5′-end at the less stable end of the duplex is more likely to be selected as guide strand whereas the other strand, with the more stable 5′-end serves as the passenger strand (Khvorova et al., 2003; Schwarz et al., 2003; **Figure 1A**). When designing small RNA duplexes for therapeutic or experimental use, highly asymmetric duplexes are desirable to reduce “off-target” effects caused by targeting of the passenger strand.

Initial understanding of the mechanism of guide strand selection came from studies of fly Ago2, where the Dicer-2/R2D2 heterodimer binds small RNA duplexes asymmetrically. R2D2 binds at the most stable end of the duplex, while Dicer-2 is positioned at the less stable end. In flies, such binding of Dicer-2/R2D2 to siRNA duplexes is a prerequisite for Ago2-RISC assembly (Liu et al., 2003; Pham et al., 2004; Tomari et al., 2004a,b). In mammals, Dicer, with the aid of its binding partners (TRBP or PACT), also binds RNA duplexes asymmetrically (Noland et al., 2011). However, in contrast to fly Ago2-RISC assembly, it has been recently shown that, Dicer is dispensable for the asymmetric assembly of fly Ago1-RISC and mammalian RISC (Kawamata et al., 2009; Betancur and Tomari, 2012; **Figure 1A**).

Although the precise mechanism of strand selection in mammals remains obscure, the crystal structures of prokaryotic and eukaryotic Ago proteins have hinted that Ago proteins themselves might be able to sense the asymmetry of the duplex. The crystal structures suggest that when an RNA duplex is loaded in Ago and the 5′-phosphate of the guide strand is docked at the phosphate-binding pocket between the MID and PIWI domains, the base pair between the first nucleotide of the guide and the complementary base of the passenger strand needs to be broken. Such structural conformation should favor the incorporation of the RNA duplex with the end that is more easily wedged (i.e., the less stable end) toward the phosphate-binding pocket (Parker et al., 2005; Wang et al., 2008a; Boland et al., 2011; Elkayam et al., 2012; Nakanishi et al., 2012; Schirle and MacRae, 2012;**Figure 1A**), which is in agreement with the well known asymmetry rule that is widely applied to the design of functional siRNAs (Khvorova et al., 2003; Schwarz et al., 2003). In the case of the fly Ago2-RISC assembly pathway, Dicer-2/R2D2 might double-check the duplex asymmetry prior to loading the duplex into Ago2.

There are additional structural requirements for RISC loading of small RNA duplexes, that we were able to reveal using an agarose native gel system (Kawamata and Tomari, 2011). According to the results, central mismatches (position 8–11 at guide strand) in small RNA duplexes are preferred for RISC loading in human Ago1–4. Duplexes containing mismatches only at non-central regions are disfavored for loading into Ago1–4. At the same time, Ago1–4 can also incorporate perfectly complementary, siRNA duplexes (Yoda et al., 2010; Gu et al., 2011; **Figure 1B**). How can human Ago proteins accommodate both siRNA duplexes and miRNA/miRNA* duplexes with apparently distinct structural properties? We have recently shown that fly Ago1 inspects the authenticity of miRNAs by utilizing multiple anchoring points (Kawamata et al., 2011). Given that fly Ago1 shares many features with mammalian Ago1–4, they should also employ a similar strategy. We envision that, while central mismatches act as an anchor to Ago, seed or 3′-mid mismatches antagonize Ago anchoring. Because most miRNA/miRNA* duplexes bear mismatches in the seed and/or 3′-mid region as well as in the central region (Kawamata et al., 2009), the destabilization effect by seed/3′-mid mismatches, which is later required for efficient slicer-independent unwinding (see below), is neutralized by the anchoring effect by the central mismatches. On the other hand, siRNA duplexes have neither the anchoring effect by central mismatches nor the debilitation effect by seed/3′-mid mismatches. This model explains how mammalian Ago1–4 selectively load authentic miRNA/miRNA* duplexes and siRNA duplexes. By contrast, in flies, Dicer-2/R2D2 acts as a gatekeeper to sort miRNA/miRNA* duplexes and siRNA duplexes into Ago1 and Ago2, respectively (Forstemann et al., 2007; Tomari et al., 2007).

It has long been known that RISC loading requires the energy of ATP (Nykanen et al., 2001; Pham et al., 2004; Tomari et al., 2004a), but the reason had been unclear. Through structural analysis of prokaryotic and eukaryotic Ago proteins, it is hypothesized that small RNA duplexes are too bulky to fit into Ago proteins (Ma et al., 2005; Parker et al., 2005; Wang et al., 2008a,b; Elkayam et al., 2012; Nakanishi et al., 2012; Schirle and MacRae, 2012). Therefore, it appears that Ago proteins require a dynamic conformational opening to accept small RNA duplexes. Recently, it was shown that the Hsc70/Hsp90 chaperone machinery acts to mediate such a conformational change of Ago proteins by using the energy of ATP hydrolysis in flies and plants (Iki et al., 2010; Iwasaki et al., 2010; Miyoshi et al., 2010). Inhibitors for Hsc70 or Hsp90 impair human RISC loading, suggesting that the chaperone machinery is also required for duplex loading in mammals (Iwasaki et al., 2010; **Figure 1B**).

## UNWINDING

After RISC loading, small RNA duplexes unwind within the Ago protein. There are two types of unwinding: slicer-dependent and slicer-independent. Slicer-dependent unwinding requires cleavage of the passenger strand by Ago proteins. In mammals, Ago2 cleaves the passenger strand of highly complementary siRNA duplexes across from position 10 and 11 of the guide (Matranga et al., 2005; Miyoshi et al., 2005; Rand et al., 2005; Leuschner et al., 2006;**Figure 1B**). On the other hand, miRNA/miRNA*-like duplexes that contain central mismatches (guide position 8–11) around the passenger cleavage site and siRNA duplexes incorporated into Ago1, 3, and 4 (that lack slicer activity) cannot be unwound by slicer-dependent unwinding. How are small RNA duplexes unwound without passenger strand cleavage? For extensively complementary duplexes, slicer-independent unwinding is intrinsically slower than slicer-dependent unwinding, because the duplexes are too stable without the passenger strand cleavage. However, slicer-independent unwinding is greatly promoted by internal mismatches. More specifically, mismatches in the seed region (guide position 2–8) and/or the 3′-mid region (guide position 12–16) facilitate unwinding in all four Ago proteins. In this way, central, seed and 3′-mid mismatches in authentic miRNA/miRNA* duplexes collaboratively promote the formation of RISC containing any of the four mammalian Ago proteins (Yoda et al., 2010; Gu et al., 2011; **Figure 1B**). In contrast to RISC loading, unwinding – both slicer-dependent and slicer-independent unwinding – does not require ATP or the chaperone machinery (Kawamata et al., 2009; Iwasaki et al., 2010; Yoda et al., 2010), suggesting that the energy required to separate the two strands is “pre-charged” upon precedent ATP-dependent loading of small RNA duplexes.

## DESIGN OF A HIGHLY SPECIFIC, ARTIFICIAL miRNA/miRNA* DUPLEX

To date, the most common RNAi triggers for experimental and therapeutic purposes are siRNA-based duplexes. However, the most abundant endogenous small RNAs in mammals are miRNAs, and our work has shown that duplexes bearing mismatches at appropriate positions exhibit miRNA-like properties and are highly efficient in both RISC loading and unwinding (Kawamata et al., 2009; Yoda et al., 2010; Betancur and Tomari, 2012). Moreover, we have previously shown that a rationally designed artificial miRNA/miRNA* duplex showed ~10-fold lower off-target effect from the passenger strand compared to a functionally asymmetric siRNA duplex, without compromising the specific silencing activity of the guide strand (Betancur and Tomari, 2012). A similar conclusion was made by Wu et al. (2011), where the effect of internal mismatches was systematically investigated for target silencing. It was shown that, especially when original siRNAs are not highly active, introducing internal mismatches can not only reduce the off-target effect but also enhance the silencing activity of the guide strand (Wu et al., 2011).

**FIGURE 2:**
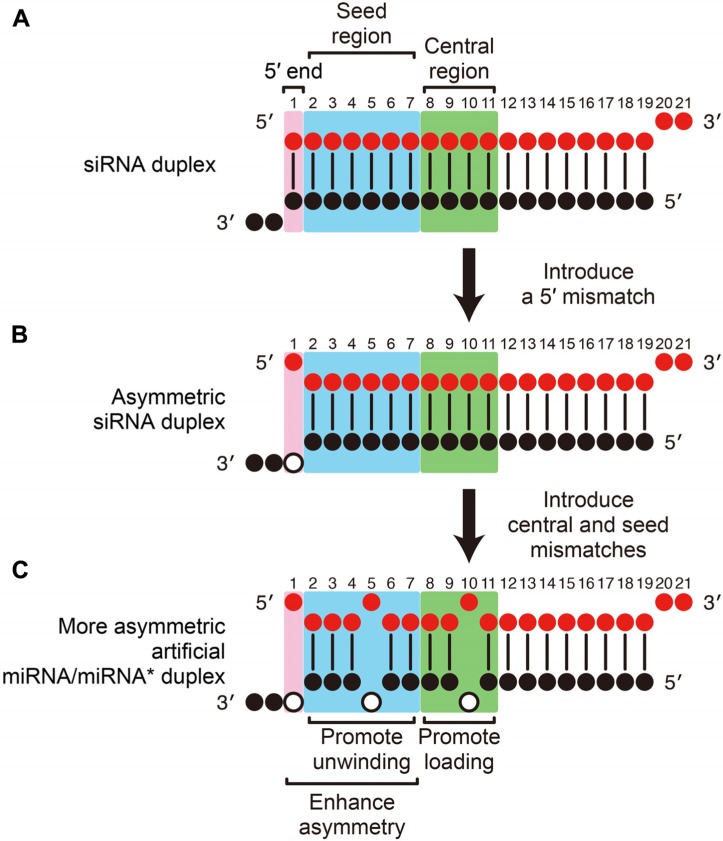
Rational design of highly efficient, artificial miRNA/miRNA* duplexes. **(A)** A conventional siRNA. **(B)** A functionally asymmetric siRNA duplex is designed by introducing a mismatch at the 5′-end of the guide strand (pink). **(C)** An artificial miRNA/miRNA* duplex is designed from the asymmetric siRNA in B by appropriately introducing internal mismatches. Mismatches in the central region (green) promote RISC loading and mismatches in the seed region (blue) promote unwinding. The functional asymmetry of the duplex is enhanced by 5′ and seed mismatches. Open circles indicate the modified nucleotides on the passenger strand to introduce mismatches.

Based on these recent findings, we can now describe a strategy to rationally design highly efficient and specific, artificial miRNA/miRNA* duplexes from conventional siRNA duplexes. First, a functionally asymmetric siRNA duplex can be designed by following the widely used thermodynamic asymmetry rule (Khvorova et al., 2003; Schwarz et al., 2003; Reynolds et al., 2004). Most conveniently, this can be achieved by introducing a mismatch at the 5′-end of the guide strand into a conventional, fully complementary siRNA, by modifying the facing nucleotide (position 19) on the passenger strand (**Figures  2A,B**). Because it has been shown that a 5′ U on the guide strand is preferred for both RISC loading and unwinding (Frank et al., 2010; Kawamata et al., 2011), and because that nucleotide is not involved in target RNA recognition (Ma et al., 2005; Parker et al., 2005), it is also reasonable to modify the 5′-end nucleotide of the guide strand to U to introduce a mismatch. Then, mismatches should be introduced in the central region (position 8–11 of the guide; to promote duplex loading) and the seed region (position 2–7 of the guide; to promote unwinding) to create a miRNA/miRNA*-like duplex (**Figure 2C**), by modifying the passenger strand sequence. Usually, introduction of a single seed mismatch and a single central mismatch is sufficient to promote duplex loading and unwinding (Yoda et al., 2010), but combination of mismatches and wobble base pairs is also possible. For example, we have successfully designed an artificial miRNA/miRNA* duplex by introducing a mismatch at position 11 and wobble base pairs at positions 9 and 10; and a mismatch at position 4 and a wobble base pair at position 3 (Yoda et al., 2010; Betancur and Tomari, 2012). Note that seed mismatches (and potentially wobble base pairs) not only promote unwinding but also enhance the thermodynamic asymmetry of the duplex, reducing the unwanted off-target effect from the passenger strand. Although mismatches in the 3′-mid region also promote unwinding, they may negatively affect the functional asymmetry of the duplex (Khvorova et al., 2003; Schwarz et al., 2003) and therefore seed mismatches are preferred for practical use. The above-described guideline, based on the mechanistic understanding of the RISC assembly pathway, is simple, efficient and cost-effective, and should be widely applicable to general gene silencing purposes.

## Conflict of Interest Statement

The authors declare that the research was conducted in the absence of any commercial or financial relationships that could be construed as a potential conflict of interest.
